# Characterizing the Interaction between Antibiotics and Humic Acid by Fluorescence Quenching Method

**DOI:** 10.3390/ijerph15071458

**Published:** 2018-07-10

**Authors:** Runze Wang, Shengke Yang, Jie Fang, Zongzhou Wang, Yangyang Chen, Dan Zhang, Chunyan Yang

**Affiliations:** Key Laboratory of Subsurface Hydrology and Ecological Effects in Arid Region, Ministry of Education, Chang’an University, Xi’an 710054, China; nighyruo@126.com (R.W.); 18792975240@163.com (J.F.); zongzhouwang@126.com (Z.W.); 18291960210@163.com (Y.C.); chzxzdd@126.com (D.Z.); 18829347435@163.com (C.Y.)

**Keywords:** antibiotic, humic acid, interaction, binding constant, fluorescence quenching

## Abstract

The method of Three-Dimensional Excitation Emission Matrix Fluorescence Spectrophotometer was used to identify the interaction mechanism between humic acid (HA) and antibiotics. The effects of antibiotic concentration, temperature, and pH on the bonding strength between HA and antibiotics were investigated. The results showed that with the increase of antibiotics concentration, the quenching effect on HA was enhanced. The quenching of HA by both oxytetracycline (OTC) and sulfadiazine (SD) is a single static quenching of the complexes, and the interaction forces are mainly a hydrogen bond and the van der Waals force. The quenching constant *K_sv_*OTC > *K_sv_*SD and the binding constant *K_b_*OTC > *K_b_*SD, indicates that HA has a more obvious quenching effect on OTC. The *K_b_* of HA by OTC ranged from 3.223 × 10^3^ to 12.784 × 10^3^ L·mol^−1^ in the range of 298 K to 313 K, while the *K_b_* of HA by SD ranged from 2.051 × 10^3^ to 5.533 × 10^3^ L·mol^−1^. With the increase of temperature, the quenching constant *K_sv_* and binding constant *K_b_* of both OTC and SD by HA gradually decrease, and the low temperature is beneficial to the interaction. The composite of OTC and HA is more stable than SD. Under neutral alkaline conditions, both OTC and SD had the strongest quenching effect on HA, and the resulting complex was the most stable. However, the *K_sv_* and *Kb* of HA by OTC were greater than SD in the pH range of the experiment and the pH effect on quenching of HA by OTC (*K_sv_*) was greater than that of SD.

## 1. Introduction

Soil organic matter (SOM) widely existing [1,2] is the sum of all natural and thermally altered biologically derived organic material found in the soil or on the soil surface irrespective of its source [1,2,3,4,5]. As SOM has various chemical compositions and functional groups [6], they play an important role in the fate and transport of organic contaminants in the environment [7,8]. Despite the long term of research, the chemical composition of SOM and its impact on contaminant transportation and migration still persist [3]. Currently, soil scientists argue that these substances do not exist in the environment as that they are extractable by a strong alkaline method [9,10,11]. Current research is still focused on simple substances such as humic acid (HA), which reveal the mechanism of the SOM interaction with contaminant and migration, and the transformation and ecological effects of the pollutants under the interaction between HA and the contaminants remain hotspots in the field of environmental biogeochemistry [6,8,12,13]. 

The key techniques in these studies include: the ion selective electrode method [14,15], voltammetry [16,17,18], potentiometric titration [19], and three-dimensional fluorescence spectroscopy [20,21]. Among these methods, fluorescence quenching turned out to be an accurate method for analyzing the interactions between contaminants and HA, as it is not limited by extraction, purification, or other physical treatments [22,23] and can directly measure changes in fluorescence intensity before and after the action of humic substances and contaminants, to obtain parameters such as the binding constant.

Antibiotics, as a typical ionic organic pollutant, often contain one or more functional groups, such as carboxyl and amino, etc., belong to the hydrophilic substances [8]. Because of their strong polarity and low volatility, antibiotics widely exist in water and soil environments [24,25,26]. The migration and transformation of antibiotics in the environment are strongly affected by humus [27,28]. Thus, people are also increasingly concerned about the environmental behavior of antibiotics with humus coexistence. Currently, the use of three-dimensional fluorescence spectroscopy to study the interaction between substances is mainly focused on the interaction of humic substances with heavy metals and hydrophobic organics [14,17,18,21]. Therefore, the use of three-dimensional fluorescence spectroscopy to study the role of humus and different types of antibiotics is of great significance for understanding the mechanism of humus and antibiotics in a water environment and its migration and transformation patterns.

In this study, humic acid was selected as the representative substance of humus, and two different types of antibiotics, oxytetracycline (OTC) and sulfadiazine (SD), were used as target contaminants. The interaction between humic acid and two antibiotics was studied by three-dimensional fluorescence spectroscopy, and the effects of antibiotics concentration, temperature, and pH and on the bonding strength between HA and antibiotics was discussed.

## 2. Materials and Methods

### 2.1. Instruments and Chemicals

**Figure 1:**
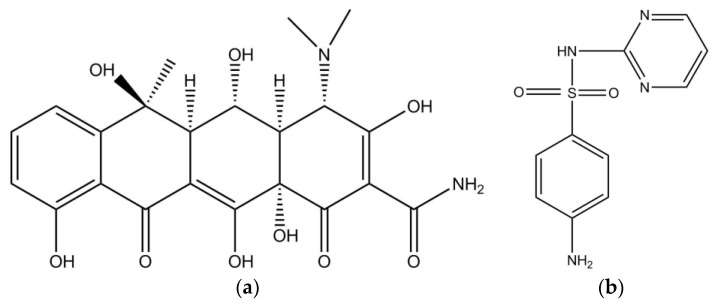
The molecular structures of (**a**) oxytetracycline (OTC) and (**b**) sulfadiazine (SD).

Oxytetracycline and sulfadiazine standard samples (purity > 95%) (Figure 1) were purchased from the German BBI company, and the Milli-Q water was used in all experiments. Other chemical reagents are of analytical grade. The main instruments are: Fluorescence spectrophotometer (Hitachi, Tokyo, Japan, F-7000), Elementar vario EL cube.

### 2.2. Preparation of Humic Acid

To purify the HA, a certain amount of biochemical reagent humic acid was weighed. The solution was first dissolved in 2% NaOH solution and then filtered through 0.45-µm filters three times. The pH of the filtrate was adjusted to less than 1.5 with 6 mol·L^−1^ hydrochloric acid. The precipitate was centrifuged at 2500 r/min for 30 min to remove the supernatant, washed with distilled water, then centrifuged for another 10 min. The precipitate was dried and stored at 277 K.

Different amounts of purified humic acid were weighed. The pH was adjusted to about 8.00 with 2% NaOH, and then filtered through a 0.22 µm membrane to obtain dissolved humic acid of different concentrations (in TOC content).

### 2.3. Experimental Methods

1 mL of 200 mg/L HA solutions were add in several 10 mL colorimetric tubes, then different amounts of 10 mg/L OTC or SD were added to keep a series of initial concentrations of 1.5, 3.0, 6.0, 7.5, 9.0 mg/L, respectively. The pH was adjusted to 8.0 with 0.1 mol·L^−1^ HCl and 0.1 mol·L^−1^ NaOH. The reaction tubes were then placed at constant temperatures of 288 K, 298 K, and 313 K respectively. Three samples were sacrificed to examine the fluorescence intensity. Also, adjusted the reaction pH to 4.0, 5.0, 6.0, 7.0, 8.0, 9.0, and 10.0, respectively, while a constant temperature of 298 K was maintained. The fluorescence intensity was tested at Ex/Em = 375 nm/460 nm.

Three-Dimensional Fluorescent scanning conditions were: excitation wavelength 300–600 nm, 5 nm increases, emission wavelength 400–700 nm, 10 nm increases, a scan rate of 1200 nm min^−1^, and the voltage amplifying of 700 V. The spectra were obtained by subtracting an ultrapure water blank spectrum, recorded in the same condition, to eliminate the Raman scatter peaks.

### 2.4. Data Analysis

The fluorescence quenching coefficients of OTC to HA were calculated using the Stern–Volmer equation [29]:

(1) $$\frac{F_{0}}{F}=1+K_{q}\zeta_{0}\left[Q\right]=1+K_{sv}\left[Q\right]$$

where *F* and *F*_0_ are the fluorescence intensity of the standard HA with and without quencher presence. [*Q*] is the quencher concentration (mg/L), *K_q_* is the quenching rate, *K_sv_* is the Stern–Volmer quenching constant, which is associated with the quencher’s ability to quench the fluorescence signal regardless of the mechanism. The *K_sv_* was determined through the linear regression of the *F*_0_/*F* values with the quencher concentration ([*Q*]). The linear slope was the *K_sv_* (L ·mol^−1^). *ζ*_0_ is the average life of the molecule when the quencher is absent, and the fluorescence lifetime of the biomacromolecule is about 10^−8^ s. Thus, the relationship of *K_sv_* and *K_q_* is

(2) $$K_{sv}=\;K_{q}\zeta_{0}$$

In addition, in static quenching process, the Stern–Volmer equation changed as

(3) $$log\frac{F_{0}-F}{F}=\;logK_{b}+\;nlog\left[Q\right]$$

where *K_b_* is the binding constant, *n* represented the amount of sorption sites. Therefore, if the appropriate quencher concentration range that provided a linear Stern–Volmer plot was selected, the binding constant and sorption site amounts could be evaluated by comparing the intercept and slope of the plot directly.

## 3. Results and Discussion

### 3.1. Spectroscopic Characteristics of HA

**Figure 2:**
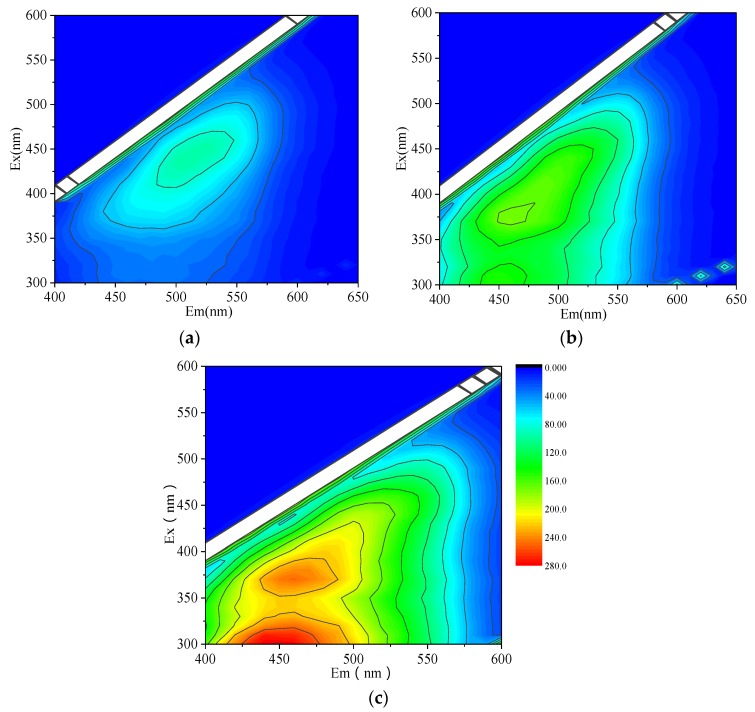
Three-Dimensional fluorescence spectrum of humic acid (HA) with different concentrations. (**a**) 4 mg/L; (**b**) 10mg/L; (**c**) 20 mg/L.

The three-dimensional fluorescence spectrums of different concentrations of HA (2, 4, 6, 10, 15, and 20 mg/L in total) were determined firstly and some typical concentrations (4, 10, and 20 mg/L) are illustrated in Figure 2. The different humus peak wavelength positions are listed in Table 1 [30,31,32]. At the concentrations of 2 and 4 mg/L, there was only one peak A appeared at Ex/Em = 410–470 nm/470–540 nm assigned to humic acid-like fluorescence. At concentrations of 6 and 10 mg/L, there was a shoulder peak with weak fluorescence intensity appeared at Ex/Em = 290–330 nm/370–450 nm besides peak A, which is assigned to fulvic acid. When the concentration increased to 15 and 20 mg/L, the shoulder peak occurred clearly and there are two distinct fluorescence peaks, peak A (Ex/Em = 350–410 nm/430–490 nm) and peak B (Ex/Em = 290–330 nm/370–450 nm). This indicated that the higher concentration (>6 mg/L) of HA contained not only humic acid-like but also ulvic acid fluorescence. Zhu et al. [33] reported the analogous results, that is, the humic acid-like and fulvic-like acid peaks were identified. However, it is known that humic acid-like substances play a major role in HA since they have a higher molecular weight, stronger aromaticity and hydrophobicity than fulvic acid. Consequently, the quenching experiment is mainly based on humic acid-like fluorescence quenching.

In addition, the fluorescence intensity gradually increased and the maximum emission of the peak A shifted slightly with a shift in the excitation wavelength for about 80 nm as the concentration of HA increased, which may be due to the possible energy transfer processes and fluorescent inner filter effects [34].

### 3.2. Quenching of HA Fluorescence by Antibiotics

**Figure 3:**
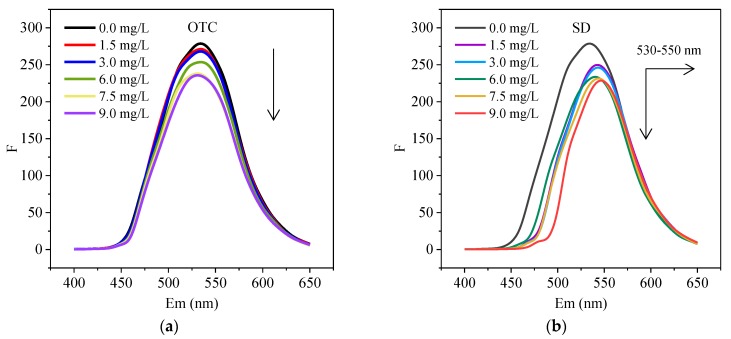
The fluorescence intensity (Ex/Em = 375 nm/400–650 nm) of HA with the presence of different concentration of (**a**) OTC and (**b**) SD.

The quenching of HA by different concentrations of OTC and SD at the temperature of 298 K and pH = 7 are shown in Figure 3. The fluorescence peak position was hardly shifted while its intensity was obviously and regularly reduced with the concentration of OTC increase, indicating that the presence of OTC can quench the HA (Figure 3a). Similarly, the fluorescence intensity of HA was reduced with the presence of SD (Figure 3b) suggesting that it can be quenched by SD. Magdalena reported the xanthine and porphyrin compounds have an aromatic structure, thus the interactions between them result in the formation of π-stacked complexes showing a red-shift of the fluorescence peak on the spectrum [35,36,37]. In this study, since SD and HA also own an aromatic structure, a red-shift of the fluorescence peak may occur through the π-stacked complex formation between SD with HA. Furthermore, some current studies found that the changes appeared in HA conformation and molecular stiffness during the fluorescence quenching of metal ions [38,39,40], because the quenching process, including the peak shift, in this study was similar to the interaction of HA with metal ions, we inferred that the HA conformation and molecular stiffness would alter in the interaction between HA and SD.

**Figure 4:**
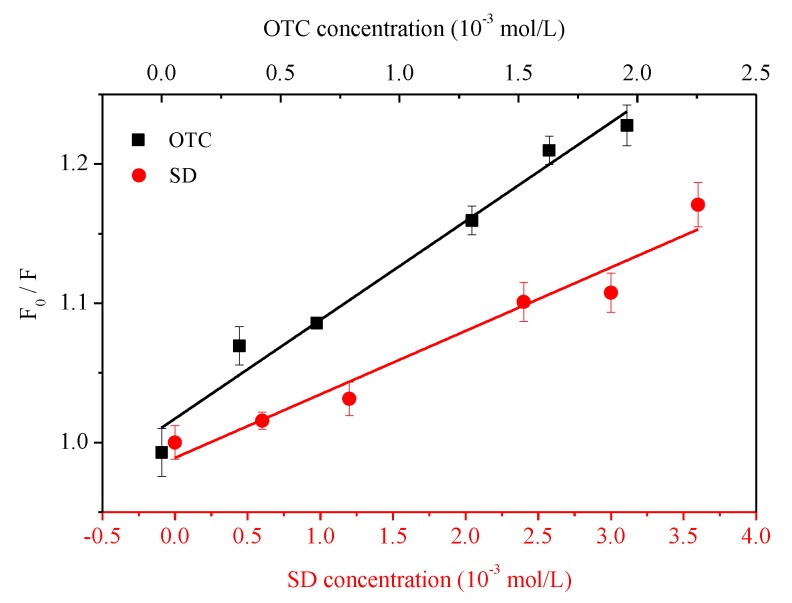
Stern–Volmer plots of OTC and SD for HA at temperature of 298 K.

The fluorescence quenching plots were fitted with Equation (1) and Figure 4 shows the linear regression of the *F*_0_/*F* values with the OTC/SD concentration ([*Q*]), thus the quenching constant could be obtained and the fitted parameters are shown in Table 2. *F*_0_/*F* with OTC or SD concentration had a significant single linear relationship, indicating that the sorption process was single quenching and the quenching intensity increased with augmented antibiotic concentration. The *K_q_* of OTC and SD were both higher than the average dynamitic quenching constant (2.0 × 10^10^ L·mol^−1^·s^−1^). Hence, the interaction between HA and OTC/SD was static quenching; i.e., the non-fluorescent complex formed by two kinds of antibiotics with HA. Wang et al. [41] suggested that the aromatic components of phenanthrene and naphthalene showed static quenching, while carboxyl groups primarily showed dynamic quenching, which was exactly in agreement with our study since the two quenchers only had an aromatic structure. In addition, the binding constant *K_b_* and the amount of binding sites could be acquired by Equation (3). The binding site number of OTC and SD with HA was 1.207 and 0.997 respectively, indicating that they formed conjugates at 1:1 proportions. However, the *K_sv_* and *K_b_* of HA by OTC were higher than SD, indicating that the quenching intensity by the former is higher than the latter, which was determined by the different structure of two kinds of antibiotics. Both of the fitted coefficients of OTC and SD were greater than 0.9, performing as the higher linear relationship between *F*_0_/*F* with OTC and SD concentration (Figure 4).

### 3.3. Temperature Effect

**Figure 5:**
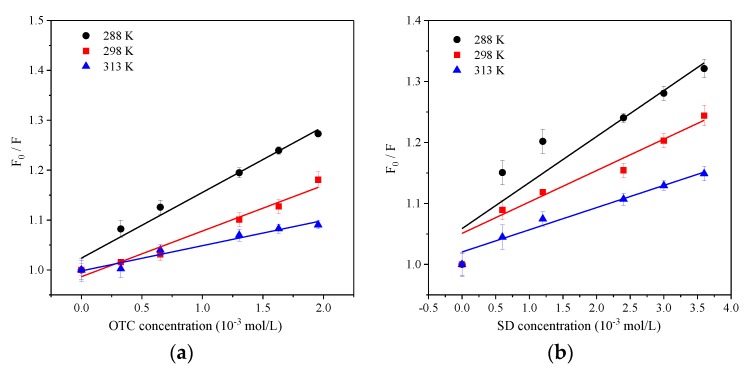
Stern–Volmer plots of fluorescence quenching of HA by antibiotics at different temperatures (**a**) OTC; (**b**) SD.

At pH 7.0, three different temperatures of 288 K, 298 K, and 313 K were set in the reaction system to investigate the effect of temperature on the interaction between OTC, SD, with HA. The Stern–Volmer plot at varied temperature is the simplest way to determine whether static or dynamic quenching is the main quenching process in the system. In the dynamic quenching process, a temperature increase leads to increasing collisional frequency and thus the *K_sv_*. In the static quenching process, on the other hand, the temperature increase tends to dissociate the fluorophore–quencher complex, resulting in a decrease of *K_sv_* [42]. In this study, the *K_sv_* values of OTC-HA and SD-HA systems decreased as the temperature increases (Figure 5), suggesting that the main quenching mechanism is the static process. The number of binding sites that OTC or SD combined with HA at the three temperatures was approximately 1, indicating that both antibiotics formed a conjugate with HA in 1:1 proportions (Table 3). The value of *K_b_*OTC ranged from 3.223 × 10^3^ to 12.784 × 10^3^ L·mol^−1^ in the range of 298 K to 313 K, while the value of *K_b_*SD ranged from 2.051 × 10^3^ to 5.533 × 10^3^ L·mol^−1^, and both gradually decrease with increasing temperature, which is consistent with the changes of *K_sv_*.

The value of *K_b_*OTC was higher than *K_b_*SD at three temperatures, that is, the complex formed between OTC and HA was more stable. On the other hand, the *K_b_* value of OTC at 313 K decreased 9.561 × 10^3^ L·mol^−1^ compared to 298 K, while the *K_b_* value of SD only decreased 3.482 × 10^3^ L·mol^−1^, indicating that the complex formed by OTC and HA is more sensitive to temperature changes. The majority temperature conditions related to a better liner relationship between the *F*_0_/*F* with antibiotics concentrations since the fitted coefficients of both were higher than 0.9, whereas a worse liner relationship was found for SD at 288 K (R^2^ < 0.9).

Thermodynamic parameters for a binding interaction were proved to be a powerful tool to determine the nature of intermolecular forces. Gibbs free energy (∆H) can be calculated by equation:

(4) ΔG=−RTlnK

The enthalpy changes (∆H) and the entropy change (ΔS) can be obtained from the intercept and slope of the deformed Van’t Hoff equation:

(5) ΔG=ΔH−TΔS

R is the ideal gas constant, 8.314 J/mol·K; T (K) is the absolute temperature and K is the partition coefficient *K_d_*. In fluorescence quenching, K is the binding constant *K_b_*.

It can be seen from Table 4 that ΔG value of OTC and SD interacted with HA less than 0, indicating that the interact process was a spontaneous reaction [43]. According to the association of the thermodynamic parameters with various interactions summarized by Ross and Subramanian [44], ΔS > 0 and ΔH > 0 indicate that the main force is the hydrophobic force, ΔS > 0 and ΔH < 0 indicate that the main force is electrostatic attraction, and ΔS < 0 and ΔH < 0 indicate that the main force is the van der Waals force or hydrogen bonds. Since both of the values are ΔH (−41.306 kJ·mol^−1^) < 0 and ΔS (−29.750 kJ·mol^−1^) < 0, the interaction forces of the two antibiotics with HA are mainly hydrogen bonds or van der Waals forces.

### 3.4. pH Effect

**Figure 6:**
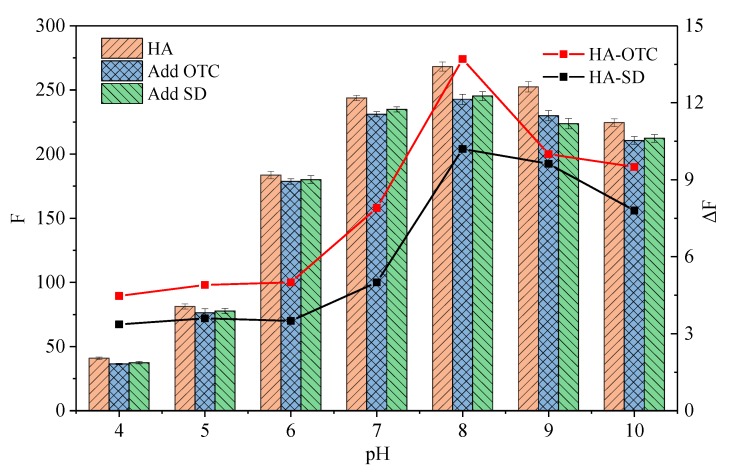
The different fluorescence intensity of the HA, HA-OTC, and HA-SD system. Δ*F* was the fluorescence intensity changes of HA with the presence OTC and SD. The concentration of antibiotics was 6 mg/L. The concentration of HA was 20 mg/L.

The different fluorescence intensities of the HA, HA-OTC, and HA-SD system at different pH values are shown in Figure 6. For HA, its fluorescence intensity increases as the pH value increases until to pH = 8.0, while a further increase of pH will slightly reduce the fluorescence intensity. Since the humic acid is a negatively charged polymer [33], its configuration is long-tail stretched due to the repulsion between the humic molecules under neutral and alkaline pH conditions [45,46], and more fluorescent groups are exposed to the solution, strengthening the fluorescence intensity [47]. While under the acidic pH conditions, the protonation of acidic groups not only quenches the fluorescence, but also leads to the polymerization of the molecules due to the strengthening of the hydrogen bond binding ability [20,45], resulting in a decrease in the solubility of HA and a sharp decrease in the fluorescence intensity [48]. The fluorescence intensity change of the HA-OTC and HA-SD system under different pH conditions is confirmed with that of HA. When the pH is 4–6, the impact of the antibiotics on the fluorescence intensity is weak, while they have a significant quenching effect on HA under neutral and alkaline pH conditions, and this quenching effect reached the strongest at pH = 8.

**Figure 7:**
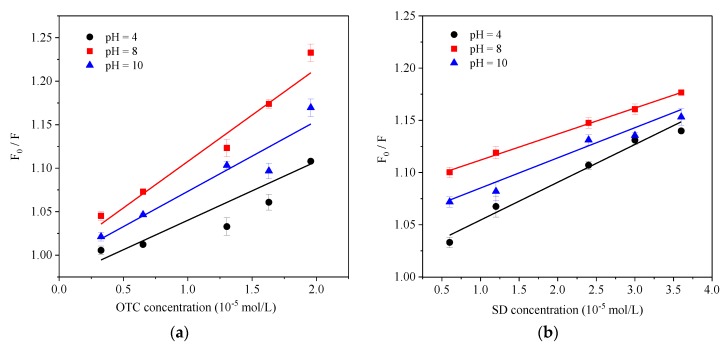
The Stern–Volmer plots of (**a**) HA-OTC and (**b**) HA-SD system at different pH values.

**Figure 8:**
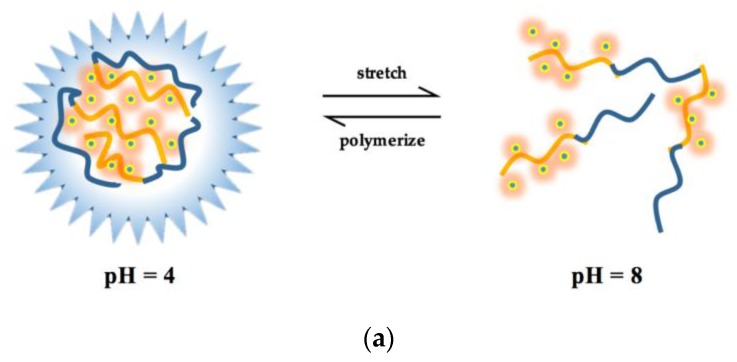

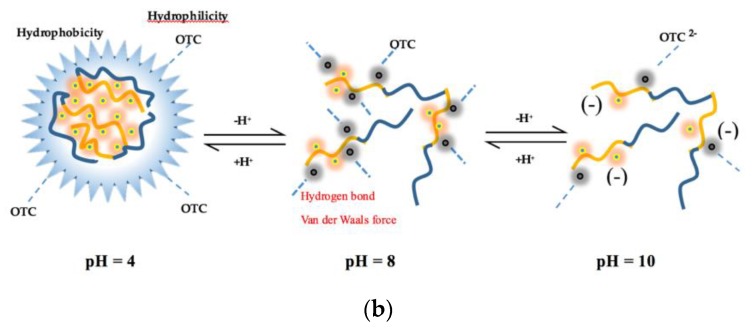
The schematic illustration of (**a**) different structures of HA under different pH conditions and (**b**) the mechanism of HA quenched by OTC.

Figure 7 shows the Stern–Volmer plots of HA-OTC and HA-SD system at different pH values and the acquired *K_sv_* and *K_b_* values are summarized in Table 5. The results show that both of the *K_sv_* values of OTC and SD increased with the pH increases until pH = 8. A further increase of pH would not relate to a higher *K_sv_*, suggesting that the fluorescence quenching of HA by OTC and SD were favorable with neutral and alkaline pH conditions. This was in agreement with the change of *K_b_* values of two antibiotics, indicating that the formed complex of HA with OTC and SD were more stable under neutral and alkaline pH conditions. HA is most likely to form a “pseudo micelle” structure under neutral and alkaline conditions [49], which is an advantageous combination of antibiotics. At an acidic pH condition, HA will agglomerate and exhibit an aggregated and bound state, forming a hydrophobic region because of charge neutralization [50], so that the interaction between HA with the hydrophilicity quencher is weak but the hydrophobicity quencher is strong on the contrary [46,51]. With the pH increases, the charge neutralization ability decreases, more hydrophilic groups are exposed, and the interaction obviously arouses [21,52] and reaches its highest at pH = 8.0. However, further increases of pH will bring about the electrostatic repulsion increases between molecules due to the deprotonation of HA [50], and OTC and SD are in the anionic form at this time, which affects the binding of HA to OTC and SD. In this study, when pH was 4 and 10, all of the fitted regression coefficients (R^2^) of OTC and SD were less than 0.9, performing as the lower linear relationship between *F*_0_/*F* with OTC and SD concentration (Figure 7) and a lower binding constant, which consisted of the mechanism. Therefore, we now present the probable mechanism of HA quenched by OTC and SD shown in Figure 8.

Different, statistically insignificant coefficients (*p*-value greater than 0.1) of OTC while significant coefficients (*p*-value less than 0.1) of SD were excluded from the analysis when pH = 4, indicating that there may not only exist static quenching for OTC under acidic pH conditions, but also dynamic quenching. When pH ranged from 4 to 10, the *K_b_*OTC value was significantly greater than that of SD (*p* < 0.05), suggesting that the binding of OTC with HA was stronger. Furthermore, the *K_b_* value of OTC changed more obviously with pH, while there was a little change of the *K_b_*SD at the acidic range, which may be due to the different combined effects of pH on the morphology of the two antibiotics and the conformation of the HA molecule [33].

## 4. Conclusions

The interaction mechanism between humic acid (HA) with two antibiotics, OTC and SD, and the interaction intensity under the influence of environmental factors was investigated.

(1) Both OTC and SD can quench HA. The quenching mechanism assigned to a single static quenching of the complex and formed conjugates at 1:1 proportions. The interaction forces were mainly hydrogen bonding or van der Waals forces; the value of *K_sv_*OTC and *K_b_*OTC were all greater than that of SD. That is, OTC had a stronger quenching effect on HA fluorescence, and the new formed complex was more stable.
(2) The influence of environmental factors on the experiment showed that: with the increase of temperature, the *K_sv_* and *K_b_* value of two antibiotics quench HA gradually decreased, and the low temperature was beneficial to the interaction. The complex formed between OTC and HA was more stable than that of SD. Under neutral alkaline conditions, both OTC and SD had the strongest quenching effect on HA, and the formed complexes were the most stable. Both the value of *K_sv_*OTC and *K_d_*OTC were greater than SD in the experiment pH. The quenching effect of OTC and HA (*K_sv_*) is significantly affected by pH, while less affected for SD.

## Figures and Tables

**Table 1:** The fluorescence peak location of different humus.

Peak	Ex	Em	Substances
peak A	350~440 nm	430~510 nm	Humic acid-like
peak B	310~360 nm	370~450 nm	Fulvic acid-like (Visible)
peak C	260~290 nm	300~350 nm	Protein like
peak D	240~270 nm	370~440 nm	Fulvic acid-like (UV)

**Table 2:** The fitted association constant of the interaction between HA, OTC, and SD.

Quencher	*K_sv_* (10^3^·L·mol^−1^)	*K_q_* (10^11^·L·mol^−1^·s^−1^)	*K_b_* (10^3^·L·mol^−1^)	*n*	R^2^
OTC	9.811	9.811	7.183	1.207	0.9708
SD	5.271	5.271	3.123	0.997	0.9470

**Table 3:** The interaction constants between antibiotics and HA at different temperatures.

Antibiotics	T (K)	*K_sv_* (10^3^·L·mol^−1^)	*K_q_* (10^11^·L·mol^−1^·s^−1^)	*K_b_* (10^3^·L·mol^−1^)	*n*	R^2^
OTC	288	13.174	13.174	12.784	0.974	0.9732
	298	9.811	9.811	7.183	1.207	0.9708
	313	5.055	5.055	3.223	1.023	0.9510
SD	288	7.513	7.513	5.533	1.104	0.8763
	298	5.271	5.271	3.123	0.997	0.9469
	313	3.163	3.163	2.051	1.045	0.9617

**Table 4:** The thermodynamic parameters.

Antibiotics	T (K)	ΔG (kJ·mol^−1^)	ΔH (kJ·mol^−1^)	ΔS (J·mol^−1^·K^−1^)
OTC	288	−22.642	−41.306	−64.807
	298	−22.000		−64.787
	313	−21.021		−64.807
SD	288	−20.636	−29.750	−31.645
	298	−19.936		−32.933
	313	−19.845		−31.645

**Table 5:** The fitted association constant of the interaction between HA, OTC and SD at different pH values.

Quencher	pH	*K_sv_* (10^3^·L·mol^−1^)	*K_q_* (10^11^·L·mol^−1^·s^−1^)	*K_b_* (10^3^·L·mol^−1^)	*n*	R^2^
OTC	4	7.282	7.282	6.430	0.944	0.5115
	8	10.916	10.916	9.872	0.914	0.9217
	10	10.363	10.363	8.890	1.288	0.7982
SD	4	3.894	3.894	3.724	1.340	0.8836
	8	4.043	4.043	4.015	0.690	0.9229
	10	3.946	3.946	3.824	0.810	0.8263
